# Establishment of Betalain-Producing Cell Line and Optimization of Pigment Production in Cell Suspension Cultures of *Celosia argentea* var. *plumosa*

**DOI:** 10.3390/plants13223225

**Published:** 2024-11-16

**Authors:** Thapagorn Sang A Roon, Poramaporn Klanrit, Poramate Klanrit, Pornthap Thanonkeo, Jirawan Apiraksakorn, Sudarat Thanonkeo, Preekamol Klanrit

**Affiliations:** 1 Graduate School, Khon Kaen University, Khon Kaen 40002, Thailand; thapagorns@kkumail.com; 2 Department of Biotechnology, Faculty of Technology, Khon Kaen University, Khon Kaen 40002, Thailand; portha@kku.ac.th (P.T.); jirapi@kku.ac.th (J.A.); 3 Research Group of Chronic Inflammatory Oral Diseases and Systemic Diseases Associated with Oral Health, Department of Oral Biomedical Sciences, Faculty of Dentistry, Khon Kaen University, Khon Kaen 40002, Thailand; porakla@kku.ac.th; 4 Department of System Biosciences and Computational Medicine, Faculty of Medicine, Khon Kaen University, Khon Kaen 40002, Thailand; porakl@kku.ac.th; 5 Cholangiocarcinoma Research Institute, Faculty of Medicine, Khon Kaen University, Khon Kaen 40002, Thailand; 6 Fermentation Research Center for Value Added Agricultural Products (FerVAAP), Khon Kaen University, Khon Kaen 40002, Thailand; 7 Walai Rukhavej Botanical Research Institute (WRBRI), Mahasarakham University, Maha Sarakham 44150, Thailand; sudarat.t@msu.ac.th

**Keywords:** antioxidant, betalain, callus, *Celosia argentea*, plant cell culture

## Abstract

The prevalence of synthetic colorants in commercial products has raised concerns regarding potential risks, including allergic reactions and carcinogenesis, associated with their use or consumption. Natural plant extracts have gained attention as potential alternatives. This research focuses on callus induction and the establishment of cell suspension cultures from *Celosia argentea* var. *plumosa*. Friable callus was successfully induced using hypocotyl explants cultured on semi-solid Murashige and Skoog (MS) medium supplemented with 1 mg/L 2,4-dichlorophenoxyacetic acid (2,4-D) and 0.1 mg/L 6-benzylaminopurine (BAP). The friable callus cell line was used to establish a suspension culture. The effects of sucrose, BAP, and tyrosine concentrations on betalain production were investigated using response surface methodology (RSM) based on central composite design (CCD). Optimal conditions (43.88 g/L sucrose, 0.15 mg/L tyrosine, and 0.77 mg/L BAP) yielded 43.87 mg/L total betalain content after 21 days, representing a threefold increase compared to the control. BAP had a significant positive impact on betalain production, and increasing BAP and sucrose concentrations generally led to higher betalain production. However, tyrosine was not a significant factor for betalain production in cell suspension cultures. Additionally, antioxidant assays showed that suspension-cultured cells (SCCs) under optimized conditions exhibited free radical scavenging activity comparable to that observed in *C. argentea* var. *plumosa* flower extract. This study indicates the potential for further research on betalain production using *C. argentea* var. *plumosa* cell cultures, which may have commercial applications.

## 1. Introduction

The predominant use of synthetic food colorings in commercial products raises concerns regarding potential health risks for consumers. These synthetic colorants offer multiple benefits, including cost-effectiveness, intense coloration, durability, and resistance to light and pH changes. However, overusing synthetic dyes could result in several health issues, including hyperactivity in sensitive children, allergic reactions, and cancers [1,2]. Therefore, the production and use of natural plant pigments as alternatives have garnered much interest due to their health benefits and the preference to avoid the potential risks associated with chemically synthesized colors. Plants are the principal producers of natural pigments, which are found in several plant parts, including roots, leaves, flowers, fruits, and seeds [3]. Examples of widely used plant pigments are chlorophylls (green), carotenoids (yellow-red, orange), anthocyanins (red, indigo, and violet), and betalains (yellow, red, and violet) [4]. These natural pigments have been proposed to have several advantages and potential applications, especially for food products [5].

Betalains are plant pigments occurring in most plant families belonging to the order Caryophyllales [6]. They represent a group of water-soluble nitrogen-containing compounds, which can be further classified into two subgroups: the red-violet betacyanins exhibiting an absorption maximum at approximately 535–540 nm, and the yellow-orange betaxanthins demonstrating an absorption maximum range of 475–480 nm [6,7,8]. There are several edible sources of betalains in many plants, such as red beetroots (*Beta vulgaris*), leafy amaranth (*Amaranthus tricolor*), cactus pear (*Opuntia ficus-indica*), dragon fruit cactus (*Hylocereus costaricensis*), quinoa (*Chenopodium quinoa*), and feathered amaranth (*Celosia argentea*) [9,10,11,12,13]. *C. argentea* var. *plumosa* has been suggested as one of the alternative sources for betalains [14]. It belongs to the Amaranthaceae family, which consists of approximately 60 species worldwide [15]. Young leaves, seedlings, young stems, and flowers are used as fresh vegetables or for cooking along with other vegetables in African and Asian countries [16]. In addition to being used as edible vegetables and ornamental plants, *C. argentea* has been widely used in traditional medicine since it has many advantageous pharmacological properties due to the accumulation of several groups of bioactive compounds, for example, phenolics, flavonoids, terpenes, and especially betalains, which give the inflorescence bright colors [15].

In recent years, betalains have demonstrated attractive bioactive properties in a number of investigations [17]. The increasing interest in betalains and their applications is driven especially by their antioxidant, antimicrobial and anticancer properties [11,17,18,19]. Betanin, a well-known example of betalains, is approved for use in defined amounts as a natural food colorant with the E number E162 (beet red), indicating that betalains have potential use in a variety of industries, including food and beverage, cosmetics, and dyes [20]. However, utilizing natural plant extracts may face certain challenges and constraints, particularly inadequate production volumes, chemical contamination, large area requirements, high labor and production costs, and extract safety concerns [21]. In addition, geographical location, climate, and crop variation can influence the production and accumulation of bioactive compounds within plants [22]. To circumvent these problems, the establishment of in vitro plant cell cultures provides a potential strategy to meet market demand for betalain production.

In vitro cultures, particularly callus and cell suspension cultures, are considered attractive biotechnological methods to obtain betalains from plants [23]. More controlled environments, including temperature, pH, photoperiod, and relative humidity within the culture system, enhance the capability of plant cells to generate and accumulate a stable quantity of bioactive compounds consistently. Moreover, this in vitro process makes it possible to produce clean and safe bioactive compounds throughout the year, regardless of weather conditions and without the need to cultivate areas [21,23]. The production of betalain compounds using plant cell cultures has been demonstrated in several betalain-accumulating plants, including *B. vulgaris*, *A. tricolor*, *Pereskia aculeata*, *H. costaricensis*, *Chenopodium quinoa*, and *C. argentea* [10,11,12,24,25,26,27]. Several essential factors potentially control cell growth and betalain production, such as carbon sources, plant growth regulators (PGRs), and amino acids as precursor molecules. Sucrose is the most suitable carbon source for plant cell cultures, as shown in the callus cultures of *C. argentea* var. *cristata* and *Bougainvillea* spp. and the suspension culture of *B. vulgaris* [27,28,29]. PGRs, specifically the types of auxin and cytokinin in an appropriate ratio, promote callus formation and the production of betalains within cells. The cytokinin, 6-benzylaminopurine (BAP) and the auxin, 2,4-dichlorophenoxyacetic acid (2,4-D) have been used in establishing callus and suspension cultures of *C. argentea* var. *plumosa*, *C. argentea* var. *cristata* and *Chenopodium quinoa*, with different concentrations [10,12,27]. Tyrosine, an aromatic amino acid, is the direct precursor of betalain biosynthesis and might be added to the culture medium to improve cell division and differentiation while also stimulating the biosynthetic pathway of betalain compounds [30]. Adding L-tyrosine enhanced betalain synthesis in the callus cultures of red dragon fruit or pitaya (*Hylocereus* spp.) by up to 1.7-fold [31,32].

Regarding the bioactive potential and promising applications of betalains from *C. argentea*, employing plant cell culture techniques to obtain the cell line yielding *C. argentea*-derived betalains extract as the new source can overcome the drawbacks of natural plant extracts. Although there are a few investigations on callus and suspension cultures of betalain-producing plants, studies on stable cell lines producing betalains in cell suspension culture, particularly in *C. argentea* var. *plumosa*, remain limited [11,12]. Notably, there is no report on how crucial factors, such as sucrose, BAP, and tyrosine, influence the total betalain content of suspension-cultured cells (SCCs). Therefore, this study aimed to establish friable callus and cell suspension cultures from *C. argentea* var. *plumosa* and investigate the optimal condition for betalain production from SCCs using response surface methodology (RSM) based on central composite design (CCD). Additionally, the growth profile, total betalain content, and antioxidant potential of the SCCs grown under the optimized conditions were investigated.

## 2. Results

### 2.1. Callus Induction, Cell Line Selection, and Proliferation

**Figure 1:**
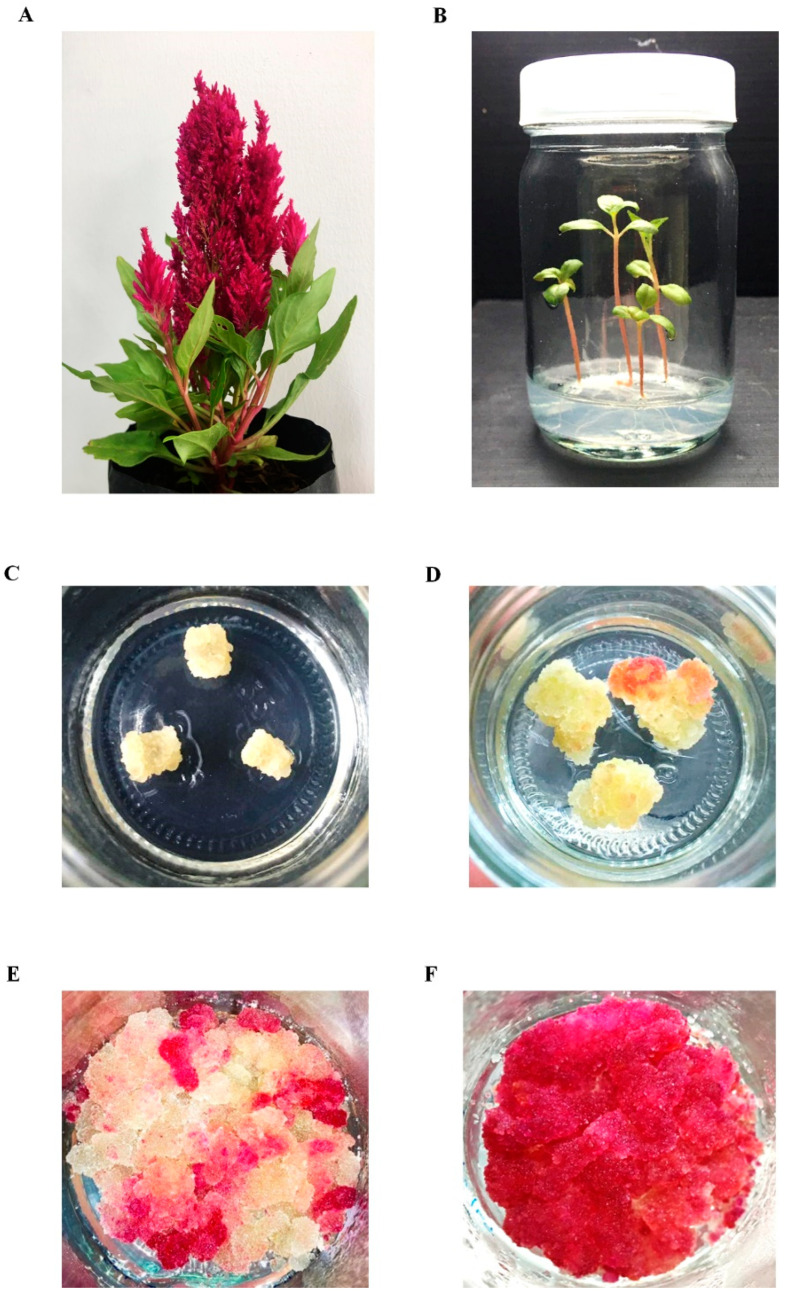
*C. argentea* var. *plumosa* plant and callus cultures: (**A**) *C. argentea* var. *plumosa* with red inflorescence; (**B**) 30-day-old seedlings; (**C**) callus initiation from explants after 2 weeks; (**D**) callus growth on callus induction medium, CIM (Murashige and Skoog (MS) medium supplemented with 1 mg/L 2,4-dichlorophenoxyacetic acid (2,4-D) and 0.1 mg/L 6-benzylaminopurine (BAP)) after 4 weeks; (**E**) proliferation of different callus colors on CIM after 15 days of starting new subculture; (**F**) red callus proliferation on CIM after 15 days of starting new subculture.

*C. argentea* var. *plumosa* is an annual herb with a compact variety that features narrow-pyramidal, plume-like flower heads ranging from 4 to 10 inches in length (Figure 1A). The experimental procedure began with seed sterilization and cultivation on Murashige and Skoog (MS) medium for 30 days (Figure 1B). Subsequently, the red-hypocotyl was excised and placed on an MS medium supplemented with various concentrations of 2,4-D and BAP. Table 1 presents the effects of 2,4-D and BAP on callus induction. After four weeks, callus formation was observed initiating from the cut edges of the explants across all treatments.

The combination of 2,4-D and BAP proved effective for callus induction, with most treatments achieving a maximum callogenesis response of 100%, except a combination of 2,4-D and BAP at 0.1:1.5, 0.1:2.0, and 2.0:0.5 mg/L yielding approximately 96.30, 92.59, and 96.29% callus induction, respectively. As anticipated, no callus development occurred in explants placed on MS medium without PGRs. The majority of the obtained calli exhibited a yellow color and compact texture (Figure 1C). Among the treatments, four yielded pink-colored calli. However, only one treatment, using 1 mg/L 2,4-D and 0.1 mg/L BAP (designated as callus induction medium, CIM), resulted in calli with semi-friable texture (Figure 1D). The pinkish-red callus was selected for further study and subcultured on semi-solid CIM every two weeks (Figure 1E). With a total of 48 subcultures, the red friable callus was obtained and proliferated well, developing a bright red color and soft texture (Figure 1F). The callus was designated as the ‘red callus line’ and was used for the establishment of cell suspension culture.

### 2.2. Establishment of Cell Suspension Cultures

**Figure 2:**
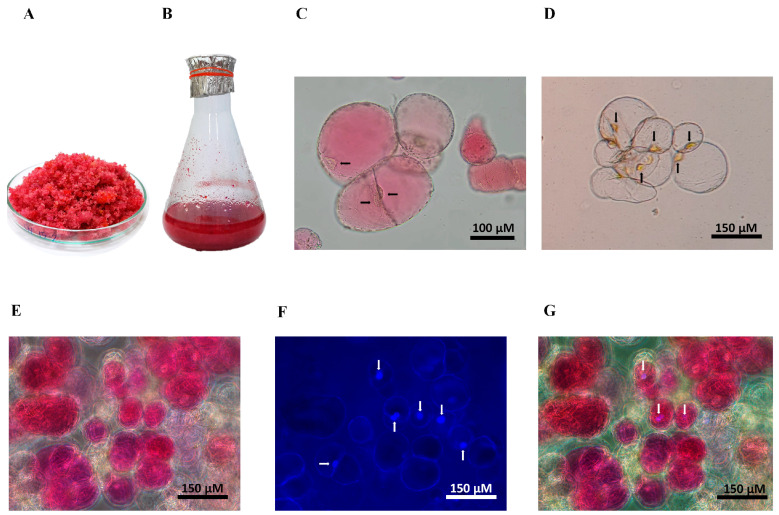
Cell suspension cultures of *C. argentea* var. *plumosa*: (**A**) friable callus from the red cell line; (**B**) suspension-cultured cells (SCCs) at 15 days of culture; (**C**) bright-field microscopy of 15-day friable pigmented callus; (**D**) bright-field microscopy of non-pigmented cells; (**E**–**G**) SCCs stained with Hoechst 33342 under fluorescent microscopy to visualize nuclei in different channels, the images were captured in the same field of view; (**E**) cells under RGB bright-field; (**F**) cells under fluorescent filter (excitation: 357 nm/emission: 447 nm); (**G**) merged RGB bright-field and fluorescence images. Arrows indicate nuclear positions, magnification = 200×, scale bar = 150 μm.

A rapidly proliferating cell line characterized by its red and friable nature was used to establish a cell suspension culture (Figure 2A). SCCs were grown in liquid CIM (MS medium supplemented with 1 mg/L 2,4-D and 0.1 mg/L BAP) (Figure 2B). Bright-field microscopy revealed both pigmented and non-pigmented cells, characterized by oval and spherical morphology with thin walls (Figure 2C,D). The pigmented cells showed visible red pigment accumulation. Nuclear visualization was achieved using fluorescence microscopy (Figure 2E–G). The growth pattern of the *C. argentea* var. *plumosa* suspension culture exhibited a pattern typical of plant cell cultures. Our preliminary results showed that cell proliferation continued until reaching peak biomass accumulation on day 15, at which point a dry weight (DW) value of 17.69 g/L was obtained. Subsequently, a decline in biomass was observed. To determine the optimal time point for SCC collection for optimization experiments and subsequent antioxidant potential analysis, total betalain content (TBC) was employed as a marker to confirm that the fast-growing cell line produced maximum betalains. The SCCs exhibited peak TBC on day 15, reaching a value of approximately 34 mg/L. Consequently, the 15-day cultured cell line was selected for preparing inoculum for suspension culture and further optimizing betalain production conditions.

### 2.3. Optimization Conditions for Betalain Production by C. argentea var. plumosa Cell Suspension Culture Using Statistical Experimental Design

The codes and actual values of the significant factors in the experimental design, including concentrations of sucrose (10 to 60 g/L), BAP (0.2 to 1 mg/L), and tyrosine (0.15 to 1 mg/L), are shown in Table 2. The experimental design matrices and the total betalain content (mg/L), as the response variable, were evaluated. Total betalain content (TBC) observed in 20 experimental runs varied from 3.41 to 38.74 mg/L, while the predicted values ranged from 11.19 to 36.89 mg/L (Table 3). To develop a quadratic polynomial regression model and a second-order polynomial Equation (1) for predicting the total betalain content (TBC, mg/L) as a function of the suspension culture variables, including sucrose concentration (A), BAP concentration (B), and tyrosine concentration (C), the TBC values were used. The final prediction equation was as follows:

TBC (mg/L) = 31.53 + 2.94A + 4.67B + 1.29C + 4.75AB − 0.5025AC + 1.00BC − 9.51A2 − 4.89B2 + 2.11C2(1)

Analysis of variance (ANOVA) was employed to assess the statistical significance of the model (Table 4). The results indicated that the established model was statistically significant, with a *p*-value of 0.0108. The R-squared (R^2^) value of the regression was 0.8132. Furthermore, the lack-of-fit test yielded a *p*-value of 0.1537, which was not statistically significant (*p*-value > 0.05). These statistical outcomes suggest that the model is reliable, indicating its capacity to predict betalain production with high accuracy. ANOVA results also revealed that the linear term of B, the interaction between A and B, and the quadratic term of A were statistically significant. These findings underscore the strong influence of sucrose and BAP on betalain production in *C. argentea* var. *plumosa* cell suspension cultures.

**Figure 3:**
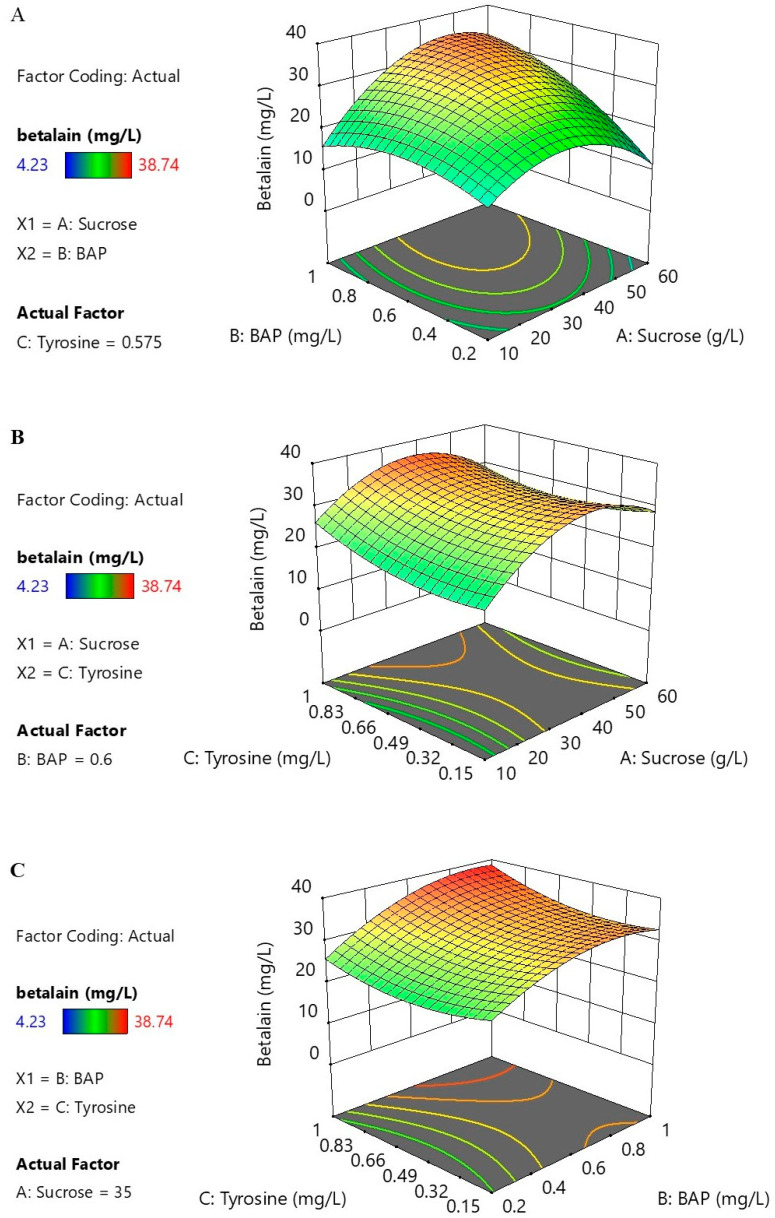
Three-dimensional response surface plots showing the effect of (**A**) sucrose, (**B**) BAP, and (**C**) tyrosine on betalain content.

The 3-dimensional response surfaces and contour plots were generated using the established model and the CCD experimental data to visualize the relationships between variables. These plots illustrated that increasing BAP and sucrose concentrations generally led to higher betalain production. However, betalain content slightly decreased when sucrose exceeded 50 g/L (Figure 3A). When BAP was held constant at its center point, maximum betalain production was achieved with tyrosine at 1 mg/L and sucrose at approximately 40 g/L. Notably, higher sucrose concentrations tended to reduce betalain production (Figure 3B). Conversely, when sucrose was fixed at its center point, increasing both BAP and tyrosine concentrations appeared to enhance betalain content (Figure 3C), suggesting a positive effect of these parameters on betalain production. The combined analysis of 3D response surfaces, contour plots, and ANOVA results indicates that BAP and sucrose are the primary factors influencing betalain production in *C. argentea* var. *plumosa* cell suspension cultures. These findings provide valuable insights for optimizing culture conditions to maximize betalain yield in this culture system.

### 2.4. Determination of Growth Profile After Optimization and Total Betalain Content (TBC)

**Figure 4:**
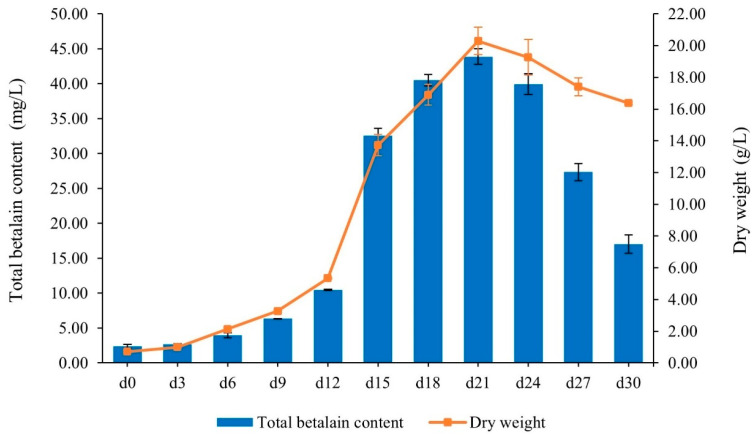
Growth profile and betalain production in *C. argentea* var. *plumosa* cell suspension cultures under optimized conditions. Bars indicate mean ± SD from triplicate experiments (n = 3).

The growth profile of *C. argentea* var. *plumosa* suspension cultures and TBC were monitored at 3-day intervals over a 30-day period following optimization. Using the medium composition derived from response surface methodology (RSM) confirmation experiments (0.15 mg/L tyrosine, 0.77 mg/L BAP, and 43.88 g/L sucrose), the results showed that cell growth continued until reaching maximum biomass accumulation on day 21, with an observed dry weight (DW) of 20.30 g/L (Figure 4). Subsequently, the biomass decreased. Concurrently, the highest TBC value of 43.87 mg/L was recorded on day 21, after which it also declined.

### 2.5. Antioxidant Activity Assays

**Figure 5:**
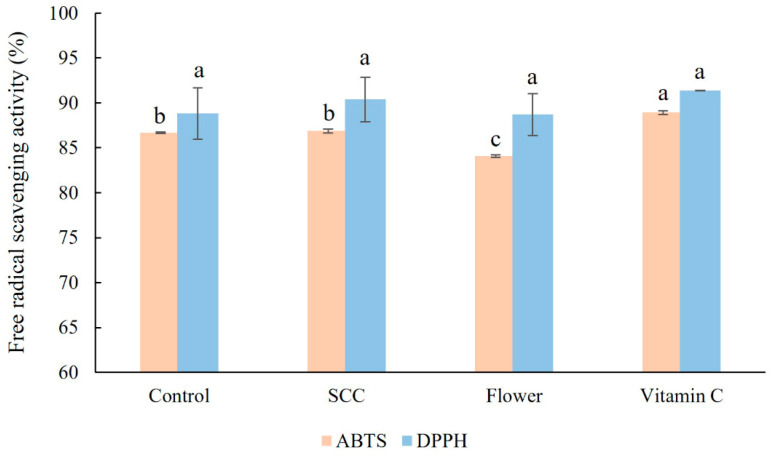
Antioxidant capacity of the SCC extracts of *C. argentea* var. *plumosa* determined by ABTS and DPPH assays. Data are presented as the means ± SD of the results of triplicate determinations. Different superscript letters within each assay indicate significant differences between samples (*p* ≤ 0.05) as determined by Duncan’s Multiple Range Test (DMRT). Control, the SCC extract from cells cultured under unoptimized conditions); SCC, the SCC extract from cells cultured under optimized conditions; Flower, the inflorescence extract, and Vitamin C (ascorbic acid).

The antioxidant capacity of inflorescence, control SCC, and optimized SCC extracts was evaluated using 2,2′-azino-bis(3-ethylbenzothiazoline-6-sulfonic acid) (ABTS) and 2,2-diphenyl-1-picrylhydrazyl (DPPH) assays (Figure 5). The ABTS scavenging activity of the optimized culture (86.88%) was comparable to the control (86.66%), with both values exceeding that of the inflorescence extract (84.07%). However, all samples exhibited lower ABTS scavenging activity than vitamin C. In the DPPH assay, all samples demonstrated similarly strong antioxidant capacity. The DPPH scavenging activity of the optimized SCC, control SCC, and inflorescence extracts were 90.39%, 88.81%, and 88.70%, respectively.

## 3. Discussion

Plant tissue and cell cultures, especially callus and cell suspension cultures, offer a promising platform for producing valuable bioactive compounds from plants for medical and pharmaceutical purposes. PGRs, especially auxins, and cytokinins, are crucial for plant cell growth and development and have been extensively utilized to stimulate callus formation in numerous plant species [10,11,12,26]. In the present investigation, a 100% callus formation response with semi-friable characteristics was achieved using red hypocotyl explants of *C. argentea* var. *plumosa*. This was accomplished by supplementing the MS medium with a combination of auxin and cytokinin (1 mg/L 2,4-D and 0.1 mg/L BAP). As expected, explants cultured on MS medium without any PGRs failed to produce a callus.

Callus induction in several betalain-accumulating plants has been demonstrated, predominantly using combinations of 2,4-D and BAP, albeit at varying concentrations. Two studies reported successful induction of red and yellow callus from *C. argentea* var. *plumosa* (the same plant species as in this work) and *Chenopodium quinoa* using 1.5 mg/L 2,4-D and 1.5 mg/L BAP [10,12]. Another study showed that callus could be induced from *C. argentea* var. *cristata* explants using MS medium supplemented with 1 mg/L 2,4-D and 1 mg/L BAP [27]. However, for callus induction in plants of other genera, such as *Hylocereus costaricensis* (red-purple dragon fruit) and *Pereskia aculeata* (leaf cactus), different PGRS including 2,4-D and picloram were used to induce pigmented friable calli [11,26]. Medium supplemented with auxin and cytokinin is necessary for callus induction and proliferation in the current investigation, as auxins and cytokinins are involved in initiating and promoting cell division, cell elongation, callus induction, and callus proliferation [33]. However, successful in vitro induction of friable callus with desired characteristics also depends on plant species. For callus induction in some species, this process requires the presence of only either auxins or cytokinin [26,34]. Considering the callus morphology with red pigment accumulation, callus friability, and the observation of the rapidly growing characteristics, the MS medium supplemented with 1 mg/L 2,4-D and 0.1 mg/L BAP (CIM) was chosen to further subculture and establish cell suspension culture of *C. argentea* var. *plumosa*. Callus cultures can also be classified as embryogenic or non-embryogenic. Embryogenic callus contains differentiated embryogenically competent cells capable of whole plant regeneration, making it valuable for developmental biology studies and transgenic research [35]. Non-embryogenic callus, characterized by homogeneous, dedifferentiated cells, is primarily utilized for secondary metabolite production, as demonstrated in this study and reported in other plant species, including *H. costaricensis* and *Chenopodium quinoa* [10,11].

A cell line selection process is essential for obtaining friable callus with specific pigment accumulation, as pigmented phenotypes are not uniformly expressed across cells from the same origin. The ability to produce pigmentation may be an intrinsic cellular characteristic, encompassing competence acquisition, differentiation, and phenotype expression [36]. In our research, the yellow callus initially developed pink coloration on its surface. Successive clonal selection of pigmented callus cells, repeated at least 48 times, enhanced red pigment accumulation. Microscopic observation subsequently revealed a predominance of cells with pinkish-red colors. This shift in metabolism likely reflects the expression of crucial genes coding for enzymes involved in betalain biosynthesis, including CYP76AD1/CYP76AD6, DOPA 4,5-dioxygenase and betanidin 5-glucosyltransferase (GT), which specifically directed towards the betacyanin pathway in red-pigmented cells [37]. While cell lines exhibiting stable, high pigment production is desirable, extended subculturing can negatively impact cell growth in both callus and suspension cultures. Previous research documented the maintenance of a stable red *Amaranthus tricolor* callus line for 24 subculture cycles [25]. By comparison, our findings demonstrate that the red cell line subcultured on CIM remains active and stable for more than three years.

Growth patterns vary among plant species, with cell suspension cultures of some plants exhibiting short growth cycles while others extend up to 30 days [11,34]. Our previous work indicated that subcultures should be performed on day 15, as biomass and total betalain content decreased thereafter due to cell mortality [38]. Transferring cells into a fresh medium at the appropriate time ensures sufficient nutrients to sustain cells and prevents the accumulation of toxic metabolic products within the culture medium [39]. In line with our current findings, suspension cultures of *C. argentea* var. *plumosa* and *Pereskia aculeata* demonstrated continuous growth, reaching maximum biomass approximately on day 15, with betalain production paralleling the increase in biomass [12,26]. Therefore, friable betalain-producing calli maintained on CIM were used as the inoculum source for establishing *C. argentea* var. *plumosa* cell suspension cultures for subsequent optimization studies.

Cell growth in suspension cultures and pigment accumulation are affected by several parameters, most importantly the medium composition. This includes carbon sources, PGRs, and precursor molecules for betalain biosynthesis. Sucrose (carbon source), BAP (PGR), and tyrosine (precursor) were selected for optimization. These parameters were collectively optimized by statistical experimental design using RSM based on CCD. This statistical experimental design method allows for the evaluation of the relative significance of several affecting factors and their interactions [40].

After optimization, we observed the highest total betalain content (TBC) of 43.87 mg/L on day 21 after culture initiation, approximately three times higher than the control (14.31 mg/L). Additionally, the highest dry weight of 20.30 g/L was obtained, which was also greater than the control (15.05 g/L). Few studies have investigated plant cell suspension culture optimization using RSM, possibly due to the challenges in obtaining consistent cell growth and the longer growth duration compared to microorganisms. Only a limited number of studies have focused on media optimization of plant cell suspension cultures for secondary metabolite production, including those in *Daucus carota* and *Centella asiatica* [40,41]. Vasilev et al. (2013) reported the optimization of *Nicotiana tabacum* cv. *BY-2* suspension line secreting the human monoclonal antibody M12, using a combination of fractional factorial designs and RSM [42]. Similar to our study, their models fit well, and the RSM plots effectively showed the interactions of different parameters of interest. In addition, the optimum composition of medium components for each plant species was identified, demonstrating the utility of RSM in plant cell culture optimization.

In in vitro plant cell culture, sucrose is relied upon heavily as a crucial carbon and energy source. As the predominant carbohydrate in phloem sap, sucrose plays a vital role in regulating various development processes, including betalain biosynthesis in *C. argentea* var. *cristata* callus culture and *B. vulgaris* cell suspension cultures [27,29,43]. The optimal sucrose concentration, however, varies among plant species. Generally, concentrations of 2–3% (*w*/*v*) support growth, while higher levels of 4–10% (*w*/*v*) could enhance product formation in plant cell cultures [43].

Different studies have employed varying sucrose concentrations depending on the plant species and culture type. For instance, *C. argentea* var. *cristata* callus cultures on semi-solid medium used 30 g/L sucrose [27], whereas *B. vulgaris* cell suspension cultures required only 10 g/L, as higher concentrations inhibited pigment production [29]. Research on *Centella asiatica* aligned with our findings, showing that sucrose positively influenced cell growth and increased dry cell weight [41]. However, their optimized conditions used a higher sucrose concentration (6.68%, *w*/*v*) compared to our study (43.88 g/L or 4.39%, *w*/*v*). Interestingly, a study on *Stenocereus queretaroensis* (a cactus) cell line demonstrated that higher sucrose concentrations (8% *w*/*v*) increased total betalain content and induced a color shift from yellow betaxanthins to red betacyanins [44].

Our RSM plots revealed that sucrose concentrations exceeding approximately 50 g/L led to decreased total betalain content. This observation is consistent with findings in Bougainvillea callus cultures, where betalain content peaked at 50 g/L sucrose before declining [28]. This reduction may be attributed to high osmotic stress caused by elevated sucrose levels, which can impair nutrient uptake and negatively impact biomass accumulation [43]. Nevertheless, our results indicate that sucrose addition up to an optimal level in the culture medium promotes betalain biosynthesis in cell suspension cultures. This finding highlights the importance of carefully optimizing sucrose concentrations to achieve a balance between cell growth and betalain production.

Numerous studies have documented the use of BAP to induce and proliferate callus and establish cell suspension cultures. BAP concentrations of approximately 1–2 mg/L have been shown to increase betalain accumulation in callus and cell suspension cultures of *C. argentea* var. *plumosa*, *C. argentea* var. *cristata*, *Amaranthus tricolor*, and *Chenopodium quinoa* [10,12,25,27]. Consistent with these findings, our study demonstrated that optimizing BAP concentration contributed positively to betalain production from *C. argentea* var. *plumosa* cell suspension cultures. However, it is crucial to note that excessive BAP concentrations can lead to cell death, as observed in *Daucus carota* and *Arabidopsis thaliana* plant cells [45]. Therefore, careful balancing of BAP levels, along with other parameters such as sucrose concentration, is essential for optimal plant growth and betalain production in cell suspension cultures [46].

The addition of exogenous tyrosine to cell suspension cultures has been proposed as a strategy for enhancing betalain synthesis and accumulation, given that tyrosine is the precursor in betalain biosynthesis [47]. However, our study demonstrated that tyrosine was not a significant factor for betalain production in cell suspension cultures. Although the RSM plots suggested that various tyrosine concentrations (either low or above 1 mg/L) could be effective, our validation experiment yielded the highest TBC value of 43.87 mg/L after 21 days using a combination of 0.15 mg/L tyrosine, 0.77 mg/L BAP, and 43.88 g/L sucrose. Notably, our initial investigations into different tyrosine levels revealed that concentrations exceeding 1 mg/L led to the formation of dark and non-viable cells, hindering the growth of suspension-cultured cells. This finding aligns with research on *H. costaricensis* callus culture, which showed that tyrosine concentrations above approximately 9 mg/L adversely affected callus proliferation [32].

These results suggest that low tyrosine concentrations are preferable for enhancing betalain production. The minimal effect of exogenous tyrosine on betalain production might be attributed to the absence or insufficiency of tyrosine hydroxylase, an enzyme that facilitates the conversion of tyrosine to L-Dopa, typically the rate-limiting step in betalain biosynthesis. Alternatively, it might result from a shortage of other essential enzymes in the betalain biosynthetic pathway within suspension-cultured cells [48,49]. Furthermore, an abundance of precursors may trigger negative feedback in the metabolic pathway, possibly leading to harmful effects on biomass and betalain accumulation in plant cells [30,50].

The current study demonstrated high antioxidant capacity in the samples, as measured by ABTS and DPPH assays. Suspension-cultured cells (SCCs) under optimized conditions exhibited free radical scavenging activity comparable to those observed in unoptimized conditions, flower extracts, and vitamin C. This similarity can be attributed to the presence of betalain compounds, known for their free radical scavenging abilities in various plant species, including *C. argentea* var. *plumosa*. The antioxidant capacity levels of the *C. argentea* var. *plumosa* SCC extracts were consistent with results reported for other betalain-accumulating plants, such as *Amaranthus* spp., *B. vulgaris*, *H. costaricensis,* and *O. ficus-indica* [11,13,51,52]. These findings suggest that the SCC extracts from *C. argentea* var. *plumosa* have significant potential for further applications, offering new opportunities for commercial betalain production.

## 4. Materials and Methods

### 4.1. Plant Material

*C. argentea* var. *plumosa* plants were grown and samples were collected in Khon Kaen Province, Thailand. The plants were identified at the Department of Biology, Faculty of Science, Khon Kaen University. The voucher specimen was deposited in the herbarium of the Department of Biology, Faculty of Science, Khon Kaen University (herbarium specimen number: KKU25556).

### 4.2. Seed Surface Sterilization

*C. argentea* var. *plumosa* seeds were surface sterilized according to Songserm et al. (2022) [34]. Seeds were initially soaked in 70% (*v*/*v*) ethanol for 5 min, followed by treatment with 0.6% (*v*/*v*) sodium hypochlorite supplemented with 2–3 drops of APSA-80 for 15 min. The seeds were then thoroughly rinsed with distilled water until all surfactant residues were removed. Surface-sterilized seeds were then placed on semi-solid Murashige and Skoog (MS) medium (PhytoTech Labs, Inc., Lenexa, KS, USA), containing 3% (*w*/*v*) sucrose and 0.6% (*w*/*v*) of the solidifying agent Gelzan^TM^ (PhytoTech Labs, Inc., Lenexa, KS, USA). The culture medium pH was adjusted to 5.8 using 1 N sodium hydroxide prior to autoclaving at 121 °C for 15 min. Seeds were cultured in 4-oz bottles containing 15 mL of the semi-solid MS medium and maintained in a culture room at 25 ± 2 °C under a 16/8 h light/dark photoperiod with cool fluorescent illumination at 45 μmol m^−2^ s^−1^. The resulting in vitro seedlings served as source materials for subsequent callus induction experiments.

### 4.3. Callus Induction

The 30-day-old red hypocotyls of *C. argentea* var. *plumosa* (1 cm in length) were used as explants for callus induction. The explant was placed on callus induction medium (CIM) with the added supplements as follows: 3% (*w*/*v*) sucrose, 0.2% (*w*/*v*) Gelzan^TM^ and followed by different concentrations of plant growth regulators (PGRs): 2,4-D (0, 0.1, 0.5, 1, 1.5 and 2 mg/L) and BAP (0, 0.1, 0.5, 1, 1.5 and 2 mg/L). The pH of the medium was adjusted to 5.8 using 1 N NaOH prior to autoclaving at 121 °C for 15 min. The control treatment is the MS medium without PGR. The cultures were kept at 25 ± 2 °C with a light intensity of 45 μmol m^−2^ s^−1^ under a 16 h light/8 h dark photoperiod regime. A factorial design was employed for a callus induction study, resulting in thirty-six treatments. Six explants were used for each treatment, as one replicate, with three replicates per treatment. All experiments were performed in triplicate. The callus induction frequency (%), color, and texture were evaluated after four weeks of culture.

### 4.4. Cell Line Selection and Proliferation

Following callus initiation, pink-colored semi-friable callus cells emerging from the explants with fast-growing characteristics were selected and subcultured on the most suitable semi-solid CIM every two weeks until the red cells with a completely friable texture and fast growth were achieved. The semi-solid CIM contains MS salts and vitamins, including 3% (*w*/*v*) sucrose, 0.2% (*w*/*v*) Gelzan^TM^, and a combination of 1 mg/L 2,4-D and 0.1 mg/L BAP. Culture conditions were controlled at 25 ± 2 °C under a cool fluorescent light intensity of 45 μmol m^−2^ s^−1^ with a photoperiod of 16 h of light/8 h of darkness.

### 4.5. Establishment of Cell Suspension Cultures and Growth Pattern After Optimization

The selected red-callus cell line with a friable texture was cultured in a liquid CIM to establish a cell suspension culture. Two grams fresh weight (FW) of friable callus was cultured in a 250 mL Erlenmeyer flask containing 100 mL of the CIM supplemented with 1 mg/L of 2,4-D and 0.1 mg/L of BAP. Cultures were maintained at 25 ± 2 °C, with a 16/8 h light/dark photoperiod, a light intensity of 45 μmol m^−2^ s^−1^, and agitated on a rotary shaker at 110 rpm. To determine the cell suspension growth profile, samples were harvested every 3 days for 30 days, with all experiments performed in triplicate [34]. The separation of the suspension-cultured cells (SCCs) from the liquid medium was achieved through vacuum filtration. Subsequently, the biomass was dried at 45 °C until a constant weight was reached, and this weight was recorded as the dry weight (DW).

Callus morphology was examined using a bright-field microscope (ZEISS Primostar 3, Oberkochen, Baden-Württemberg, Germany). Nuclear visualization was performed using Hoechst 33342 nucleic acid stain (10 μg/mL, ready-to-use). Briefly, SCCs were incubated with 100 μL of Hoechst 33342 dye for 5 min in darkness. The cells were then washed three times with MS liquid medium and observed using an EVOS^TM^ M5000 imaging system (Thermo Fisher Scientific, Waltham, MA, USA).

### 4.6. Optimization Conditions for Betalain Production by C. argentea var. plumosa Cell Suspension Culture Using Statistical Experimental Design

In this study, sucrose, tyrosine, and BAP were investigated for their effects on cell biomass accumulation and betalain production. The central composite design (CCD) approach-based response surface methodology (RSM) was applied to statistically analyze the effects of these variables. The codes and actual values of these independent factors were presented. All experiments were performed in triplicate using 250 mL Erlenmeyer flasks, each containing 100 mL of liquid MS medium (pH 5.8) with all factors. SCCs were cultured for 15 days. Total betalain content (TBC) and cell dry weight (DW) were set as the response variables. The Design-Expert^®^ version 13 software (trial version) (State-Ease, Inc., Minneapolis, MN, USA, www.statease.com) was used to generate the experimental designs and the 3-dimensional response surface and contour plots. Analysis of variance (ANOVA) was carried out to estimate statistically significant parameters. A validation experiment was then carried out using the optimized conditions derived from the response surface analysis.

### 4.7. Preparation of Extract

In this study, water was used as an extractor. First, red inflorescence (from 3-month-old plants) and SCCs of *C. argentea* var. *plumosa* were dried at 45 °C in a hot air oven (Nüve FN500, Akyurt, Ankara, Turkey) for 48 h and then ground into a fine powder. Five grams of inflorescence and callus powder were each mixed with 100 mL distilled water and incubated at 150 rpm, 50 °C for 1 h. The supernatant was filtered using a tea filter and then lyophilized (Christ, Osterode, Germany) until a fine powder was obtained. Ten milligrams of the resulting betalain powder were dissolved in 1 mL of distilled water. The solution was then filtered through a 0.2-µm pore size filter. Finally, the extracts were used for the analysis of total betalain content and antioxidant activity.

### 4.8. Determination of Total Betalain Content (TBC)

The total betalain content (betaxanthin; BX and betacyanin; BC) was measured using a microplate reader (Infinite^®^ 200 PRO, Tecan Trading AG, Männedorf, Switzerland), using a modified method of Winson et al. (2020) [11]. Initially, 350 μL (10 mg/mL) of extracts were quantified at detection wavelengths of 483 nm and 535 nm, corresponding to the maximum absorption of BX and BC, respectively. The total betalain content was expressed as the combined amount of BX and BC. To calculate the total betalain content, the following Equation (2) is used, and then the obtained values were converted into the unit mg/L:

$$BX\;or\;BC\;(mg/g)\;=\;\frac{A\times DF\times MW\times V}{\varepsilon \times L\times M}$$

Total betalain content (mg/g) = BX + BC(2)

where A is the absorption maximum determined at 483 nm and 535 nm for betaxanthin (BX) and betacyanin (BC), respectively. DF is the dilution factor. MW is the molecular weight of BX (308 g/mol) and BC (550 g/mol), respectively. V is the total volume of the sample (mL). ε is the extinction coefficient of BX (48,000 L mol^−1^ cm^−1^) and BC (60,000 L mol^−1^ cm^−1^). L is 1 cm path length (with the path length correction performed), and M is the dried mass of the sample (g).

### 4.9. Antioxidant Activity Assays

Antioxidant activity was determined using the 2,2′-azinobis-(3-ethylbenzothiazoline-6-sulfonate) (ABTS) radical scavenging assay, following the procedures described by Smeriglio et al. (2017) [53]. The ABTS•+ radical solution (2,2-azino-bis (3-ethylbenzthiazoline-6-sulforic acid), which is green in color, loses its hue in the presence of antioxidant compounds. To generate the ABTS•+ radical solution, 4.3 mM potassium persulfate (K_2_S_2_O_8_, *w/v* in water) was mixed with 1.8 mM ABTS solution at a 1:5 ratio (*v*/*v*). This mixture was then incubated for 1 h at 25 °C in darkness. Prior to sample testing, the ABTS•+ radical solution was diluted with distilled water to achieve an absorbance of 0.7 ± 0.02 at 734 nm. For the assay, 10 μL of each extract (10 mg/mL) was added to 200 μL of the diluted ABTS•+ solution. These samples were incubated in the dark for 6 min, after which the absorbance at 734 nm was measured using a microplate reader. Trolox was employed as a reference compound for this assay.

The 2,2-diphenyl-1-picryl hydrazyl (DPPH) assay was conducted following the method of Shalaby and Shanab [54] with slight modifications. Ten milligrams of inflorescence and SCC extracts were dissolved in 1 mL of distilled water. A 0.1 mM DPPH radical solution was prepared in methanol. In a 96-well plate, 100 μL of extract (or 10 mg/mL ascorbic acid in absolute methanol) was combined with 100 μL of DPPH reagent. The mixture was incubated in darkness for 30 min at 25 °C, after which the absorbance was measured at 515 nm using a microplate reader.

The antioxidant capacity of the extracts was quantified using both ABTS and DPPH assays. The percentage inhibition of radicals, expressed as scavenging activity, was calculated using the following Equation (3):

% scavenging activity = [(A_control_ − A_sample_)/(A_control_)] × 100 (3)

where A_control_ is the absorbance of the diluted ABTS•+ or DPPH radical without sample, and A_sample_ is the absorbance of the reaction mixture containing both the diluted radical (ABTS•+ or DPPH) and the sample.

## Figures and Tables

**Table 1:** Callus induction from in vitro derived hypocotyl explants of *C. argentea* var. *plumosa* after 4 weeks of culture on Murashige and Skoog (MS) medium supplemented with different concentrations and combinations of 2,4-D and BAP.

2,4-D (mg/L)	BAP (mg/L)	Callus Induction * (%)	Callus Color	Callus Texture
0	0	0.00 ± 0.00 ^c^	-	-
0	0.1	100.00 ± 0.00 ^a^	Yellow	Compact
0	0.5	100.00 ± 0.00 ^a^	Yellow/pink	Compact
0	1	100.00 ± 0.00 ^a^	Yellow/pink	Compact
0	1.5	100.00 ± 0.00 ^a^	Yellow	Compact
0	2	100.00 ± 0.00 ^a^	Yellow	Compact
0.1	0	100.00 ± 0.00 ^a^	Bright yellow	Compact
0.1	0.1	100.00 ± 0.00 ^a^	Yellow/pink	Compact
0.1	0.5	100.00 ± 0.00 ^a^	Yellow	Compact
0.1	1	100.00 ± 0.00 ^a^	Yellow	Compact
0.1	1.5	96.30 ± 6.42 ^ab^	Yellow	Compact
0.1	2	92.59 ± 12.83 ^ab^	Yellow	Compact
0.5	0	100.00 ± 0.00 ^a^	Bright yellow	Compact
0.5	0.1	100.00 ± 0.00 ^a^	Yellow	Compact
0.5	0.5	100.00 ± 0.00 ^a^	Yellow	Compact
0.5	1	100.00 ± 0.00 ^a^	Bright yellow/yellow	Compact
0.5	1.5	100.00 ± 0.00 ^a^	Yellow	Compact
0.5	2	100.00 ± 0.00 ^a^	Bright yellow/yellow	Compact
1	0	100.00 ± 0.00 ^a^	Bright yellow	Compact
1	0.1	100.00 ± 0.00 ^a^	Pink/yellow	Semi friable
1	0.5	100.00 ± 0.00 ^a^	Bright yellow/yellow	Compact
1	1	100.00 ± 0.00 ^a^	Yellow	Compact
1	1.5	100.00 ± 0.00 ^a^	Bright yellow/yellow	Compact
1	2	100.00 ± 0.00 ^a^	Bright yellow/yellow	Compact
1.5	0	100.00 ± 0.00 ^a^	Bright yellow	Compact
1.5	0.1	100.00 ± 0.00 ^a^	Bright yellow	Compact
1.5	0.5	100.00 ± 0.00 ^a^	Bright yellow	Compact
1.5	1	100.00 ± 0.00 ^a^	Bright yellow/yellow	Compact
1.5	1.5	100.00 ± 0.00 ^a^	Bright yellow	Compact
1.5	2	100.00 ± 0.00 ^a^	Yellow	Compact
2	0	100.00 ± 0.00 ^a^	Bright yellow	Compact
2	0.1	100.00 ± 0.00 ^a^	Bright yellow/yellow	Compact
2	0.5	96.29 ± 6.42 ^ab^	Bright yellow	Compact
2	1	100.00 ± 0.00 ^a^	Bright yellow	Compact
2	1.5	100.00 ± 0.00 ^a^	Bright yellow	Compact
2	2	100.00 ± 0.00 ^a^	Bright yellow	Compact

* The data are expressed as the average ± standard deviation (SD), derived from three independent experiments. Statistical analysis was performed using one-way analysis of variance (ANOVA) followed by Duncan’s multiple range test (DMRT). Within each column, mean values marked with different superscripts (a, b, or c) indicate statistically significant differences at a confidence level of 95% (*p* < 0.05).

**Table 2:** Codes and actual values of the independent factors for central composite design (CCD) on betalain production from *C. argentea* var. *plumosa*.

Code	Factor	Unit	Level				
			−1.31	−1	0	+1	+1.31
A	Sucrose	g/L	2.098	10.000	35.000	60.000	67.902
B	BAP	mg/L	0.074	0.200	0.600	1.000	1.126
C	Tyrosine	mg/L	0.016	0.150	0.570	1.000	1.134

**Table 3:** The CCD matrix for betalain production using *C. argentea* var. *plumosa*.

Std	Run	Sucrose (g/L)	BAP (mg/L)	Tyrosine (mg/L)	Betalain Content (mg/L)	
					Predicted	Observed
6	1	60.00	0.20	1.00	12.55	13.66
11	2	35.00	0.07	0.57	16.93	9.27
7	3	10.00	1.00	1.00	19.02	20.21
20	4	35.00	0.60	0.57	31.53	32.48
1	5	10.00	0.20	0.15	15.61	18.51
18	6	35.00	0.60	0.57	31.53	31.56
9	7	2.09	0.60	0.55	11.19	3.41
2	8	60.00	0.20	0.15	12.99	13.62
4	9	60.00	1.00	0.15	29.82	25.33
19	10	35.00	0.60	0.57	31.53	35.64
16	11	35.00	0.60	0.57	31.53	38.74
12	12	35.00	1.12	0.57	29.21	32.65
14	13	35.00	0.60	1.13	36.89	31.82
10	14	67.90	0.60	0.57	18.93	22.49
13	15	35.00	0.60	0.01	33.50	34.35
15	16	35.00	0.60	0.57	31.53	26.59
3	17	10.00	1.00	0.15	13.43	14.14
8	18	60.00	1.00	1.00	33.40	32.33
17	19	35.00	0.60	0.57	31.53	29.53
5	20	10.00	0.20	1.00	17.19	23.50

**Table 4:** Analysis of variance (ANOVA) and results of regression analysis of the CCD on betalain production using *C. argentea* var. *plumosa*.

Source	Sum of Squares	df	Mean Square	F-Value	*p*-Value	Note
Model	1486.93	9	165.21	4.84	0.0108	significant
A-sucrose	99.01	1	99.01	2.90	0.1194	
B-BAP	249.58	1	249.58	7.31	0.0222	
C-tyrosine	19.03	1	19.03	0.55	0.4725	
AB	180.88	1	180.88	5.30	0.0441	
AC	2.02	1	2.02	0.05	0.8127	
BC	8.08	1	8.08	0.23	0.6371	
A^2^	630.27	1	630.27	18.46	0.0016	
B^2^	166.35	1	166.35	4.87	0.0518	
C^2^	31.15	1	31.15	0.91	0.3621	
Residual	341.44	10	34.14			
Lack of Fit	248.04	5	49.61	2.66	0.1537	not significant
Pure Error	93.39	5	18.68			
Cor Total	1828.37	19				
R-Squared (R^2^) = 0.8132						

## Data Availability

The original contributions presented in the study are included in the article, further inquiries can be directed to the corresponding author/s.
